# Engineering Bacterial Surface Displayed Human Norovirus Capsid Proteins: A Novel System to Explore Interaction Between Norovirus and Ligands

**DOI:** 10.3389/fmicb.2015.01448

**Published:** 2015-12-22

**Authors:** Mengya Niu, Qianqian Yu, Peng Tian, Zhiyong Gao, Dapeng Wang, Xianming Shi

**Affiliations:** ^1^Department of Food Science and Technology, MOST-USDA Joint Research Center for Food Safety, School of Agriculture and Biology, Shanghai Jiao Tong UniversityShanghai, China; ^2^Produce Safety and Microbiology Research Unit, Western Regional Research Center, Agricultural Research Service, United States Department of AgricultureAlbany, CA, USA; ^3^Beijing Center for Diseases Prevention and ControlBeijing, China

**Keywords:** human noroviruses, cell surface display, receptor, ice nucleation protein, histo-blood group antigens

## Abstract

Human noroviruses (HuNoVs) are major contributors to acute nonbacterial gastroenteritis outbreaks. Many aspects of HuNoVs are poorly understood due to both the current inability to culture HuNoVs, and the lack of efficient small animal models. Surrogates for HuNoVs, such as recombinant viral like particles (VLPs) expressed in eukaryotic system or P particles expressed in prokaryotic system, have been used for studies in immunology and interaction between the virus and its receptors. However, it is difficult to use VLPs or P particles to collect or isolate potential ligands binding to these recombinant capsid proteins. In this study, a new strategy was used to collect HuNoVs binding ligands through the use of ice nucleation protein (INP) to display recombinant capsid proteins of HuNoVs on bacterial surfaces. The viral protein-ligand complex could be easily separated by a low speed centrifugation step. This system was also used to explore interaction between recombinant capsid proteins of HuNoVs and their receptors. In this system, the VP1 capsid encoding gene (ORF2) and the protruding domain (P domain) encoding gene (3′ terminal fragment of ORF2) of HuNoVs GI.1 and GII.4 were fused with 5′ terminal fragment of INP encoding gene (*inaQn*). The results demonstrated that the recombinant VP1 and P domains of HuNoVs were expressed and anchored on the surface of *Escherichia coli* BL21 cells after the bacteria were transformed with the corresponding plasmids. Both cell surface displayed VP1 and P domains could be recognized by HuNoVs specific antibodies and interact with the viral histo-blood group antigens receptors. In both cases, displayed P domains had better binding abilities than VP1. This new strategy of using displayed HuNoVs capsid proteins on the bacterial surface could be utilized to separate HuNoVs binding components from complex samples, to investigate interaction between the virus and its receptors, as well as to develop an oral vaccine for HuNoVs.

## Introduction

Human noroviruses (HuNoVs) are one of major nonbacterial pathogens for foodborne gastroenteritis (Atmar and Estes, 2006). HuNoVs belong to the family of *Caliciviridae*. The single-stranded, positive-sensed RNA viral genome can be divided into three open reading frames (ORFs) (Jiang et al., 1993). The ORF2 encodes the major structural protein (VP1) which have two domains: the shell (S) and the protruding (P) domain (Zheng et al., 2006). The viral capsid is composed of 180 capsid protein monomers organized into 90 dimers (Tan et al., 2008).

Due to a lack of cultivable system or animal models, surrogate viruses such as feline calicivirus (FCV), murine norovirus (MNV), and Tulane virus (TV) are often used to study the fundamental biology of the viruses such as viral replication pattern and mechanism of infection (Farkas, 2015). Recombinant human norovirus capsid proteins expressed either by eukaryotic or prokaryotic systems are often used for studies for immunogenicity, diagnosis assays, and host-receptor interaction (Gray et al., 1993; Green et al., 1993; Hutson et al., 2003; Huang et al., 2005). The virus-like particles (VLPs), spontaneously formed in a recombinant baculovirus system, have morphological and antigenical similarities to viral particles (Jiang et al., 1992). However, large production of recombinant proteins is still difficult owing to low protein yield and lack of VLPs. Tan et al. applied *E. coli* expression system to produce recombinant norovirus capsid proteins (Tan et al., 2004). They demonstrated that the *E. coli*-expressed capsid proteins maintained the same antigenicity and receptor binding specificity as those of the baculovirus-expressed VLPs, although the *E. coli*-expressed capsid proteins did not form VLPs. Tan et al. further demonstrated that a smaller particle (P particle) could form, which was expressed in *E. coli* (Tan et al., 2004). The P particles expressed *in vitro* is an octahedral nanoparticle formed by 24 copies of P monomers, most likely organized into 12 P dimers. These P particles are easily produced in *E. coli*, extremely stable, and highly immunogenic (Tan and Jiang, 2005b). However, one disadvantage of the bacterial system for expression of recombinant HuNoVs capsid proteins is that the expressed capsid proteins cannot be purified easily. After the fusion tag is removed, gel filtration and anion-exchange chromatography are required for purification of recombinant HuNoVs capsid proteins (Tan et al., 2004). Another disadvantage is that VP1 and P particles expressed by bacteria are either soluble proteins (VP1) or small particles. Therefore, it will be hard to either collect or isolate the viral capsid protein-ligand complex.

Bacterial ice nucleation proteins (INPs) are a family of proteins that enable Gram-negative bacteria to promote crystal formation at relatively high temperatures (Kawahara, 2002). Different INP coding genes from *Pseudomonas syringae, Erwinia herbicola*, and *Xanthomonas campestris* were well characterized (Wolber et al., 1986; Schmid et al., 1997; Jung et al., 1998a; Li et al., 2012). INP composes three distinct structural domains: an N-terminal domain, a C-terminal domain and a highly repetitive central domain (Shimazu et al., 2001). So far, INPs have been applied in various perfect bacterial cell surface display systems, including host cells of *E. coli* (Jung et al., 1998b; Kwak et al., 1999; Li et al., 2009), *Salmonella typhi* (Lee et al., 2000), *Vibrio anguillarum* (Xu et al., 2008), *P. syringae* (Shimazu et al., 2003). By transformation of bacteria with the gene encoding a fusion target protein with the anchoring motifs of INP, the target protein could be directly displayed on the surface of the bacteria (Kim and Yoo, 1999; Kwak et al., 1999; Cochet and Widehem, 2000). It was reported that the N-terminal domain of InaQ (named as InaQN) is responsible for the transmembrane transport and membrane-binding activity of INP (Li et al., 2012).

In order to solve the problem of collecting ligands binding to viral capsid proteins, recombinant HuNoVs capsid proteins were displayed on the surface of bacteria with the help of InaQN. It was reported that histo-blood group antigens (HBGAs) have been recognized as receptors for HuNoVs (Hutson et al., 2003; Tan and Jiang, 2005a). Therefore, in this study, Type III porcine gastric mucin (PGM) containing HBGAs (Tian et al., 2008) was used to evaluate the binding efficacy between the viral receptors and displayed HuNoVs VP1s and P domains.

## Materials and methods

### Bacterial strains and plasmids

*E. coli* DH5α and BL21 (ThermoFisher, Shanghai, China) were used as competent cells for recombinant plasmid construction and protein expression. Plasmid pMD19-T (TaKaRa, Dalian, China) inserted with different gene fragments was used for subcloning into the prokaryotic expression plasmid pET-28a (ThermoFisher, Shanghai, China). pCR-TORO/GI.1-ORF2+3 plasmid with inserted gene of HuNoV GI.1 ORF2 was kindly provided by Dr. Peng Tian (PSMRU, WRRC, USDA, CA, USA). pTrc-HisC-inaQ plasmid was kindly provided by Prof. Lin Li (State Key Laboratory of Agricultural Microbiology, Huazhong Agricultural University, Wuhan, China).

### Cloning of *inaQn*, HuNoVs GI.1/GII.4 ORF2 and P domain coding fragments

The coding sequence of InaQN domain (*inaQn*) was subcloned from plasmid pTrc-HisC-inaQ according to the previous report (Li et al., 2012).

HuNoV GI.1 ORF2 and its 3′ terminal fragments (named P) were amplified from recombinant plasmid pCR-TORO/GI.1-ORF2+3. A 1584 bp fragment for HuNoV GI.1 ORF2 (GenBank No. M87661) and a 909 bp fragment for HuNoV GI.1 ORF2 3′ terminal were amplified by PCR. Each 20.0 μL PCR reaction including 2.0 μL 10 × PCR Buffer, 1.5 μL 25 mmol/L Mg^2+^, 1 U *Taq* E (ThemoFisher, Shanghai, China), 1.0 μL 10 mmol/L dNTPs, 1.0 μL 10 mmol/L of upstream and downstream primers respectively, 2.0 μL plasmid and double distilled water (dd H_2_O). Thermal cycling condition consists of initial denaturation at 95°C for 5 min, 35 cycles of template denaturation at 95°C for 30 s, primer annealing at 52°C for 30 s, primer extension at 72°C for 1 min 40 s, and final extension at 72°C for 10 min.

Genomic RNA of HuNoV GII.4 strain was extracted by Trizol Kit (ThemoFisher, Shanghai, China) from clinical gastroenteritis samples provided by Beijing Center for Disease Control and Prevention, China. A 1623 bp fragment (GenBank No. KM114291) for HuNoV GII.4 ORF2 was amplified by two-step RT-PCR kit (TaKaRa, Dalian, China) in accordance with manufacturer's protocol. The 3′ terminal fragment (948 bp) of ORF2 was amplified according to previous reports (Wang et al., 2008).

All primers used in this study were listed in Table 1. PCR products were analyzed by electrophoresis on 1.5% agarose gels and target bands were recycled using AxyPrep DNA gel extraction kit (Corning, Shanghai, China). The recovered nucleic acid fragments were inserted into plasmid pMD19-T, named pMD19-ORF2(GI.1), pMD19-ORF2(GII.4), pMD19-P(GI.1), and pMD19-P(GII.4), respectively. The recombinant plasmids were sequenced by Shanghai Majorbio Bio-pharm Technology Co., Ltd.

**Table 1:** **Sequences of (RT-) PCR primers**.

**Gene fragment**	**Primer**	**Restriction enzyme site**	**Sequence(5′-3′)**	**Amplicon size (bp)**
GI.1 ORF2	GI.1F	*Bgl* II	AGATCTATGATGATGGCGTCTAAGG	1584
	GI.1R	*Hin*d III	AAGCTTACAGACCAAGCCTACCTC	
GI.1 P domain	GI.1 P-F	*Bgl* II	AGATCTCAGAAAACCAGGCCCTTC	909
	GI.1 P-R	*Hin*d III	AAGCTTCTAAAGCCAAGCCTTACG	
GII.4 ORF2	GII.4F	*Bgl* II	GGAAGATCTATGAAGATGGCGTCG	1623
	GII.4R	*Eco*R I	CCGGAATTCTTATAAAGCACGTCTG	
GII.4 P domain	GII.4 P-F	*Bgl* II	AGATCTTCAAGAACTAAACCATTCTC	948
	GII.4 P-R	*Eco*R I	GAATTCTTATAGTGCACGCCTACGCC	

After restriction enzymes digestion (Table 2), *inaQn* fragment and ORF2 or ORF2 3′ terminal fragments from GI.1 or GII.4 were inserted into pET-28a to create recombinant plasmids pET28a-inaQn-ORF2(GI.1), pET28a-inaQn-ORF2(GII.4), pET28a-inaQn-P(GI.1), and pET28a-inaQn-P(GII.4), respectively. In addition, pET28a-ORF2(GI.1), pET28a-ORF2(GII.4), pET28a-P(GI.1), and pET28a-P(GII.4) without the *inaQn* gene were also constructed as negative controls. All recombinant plasmids were confirmed by sequencing by Shanghai Majorbio Bio-pharm Technology Co., Ltd.

**Table 2:** **Recombinant plasmids with restriction enzyme sites**.

**Recombinant plasmids**	**Restriction enzyme sites**	**The length of nucleic acid fragments (bp)**
pET28a-1	*Nco* I & *Hin*d III	5246
pET28a-2	*Nco* I & *Eco*R I	5265
pMD19-ORF2(GI.1)	*Bgl* II & *Hin*d III	1584 (GI.1 ORF2)
pMD19-ORF2(GII.4)	*Bgl* II & *Eco*R I	1623 (GII.4 ORF2)
pMD19-P(GI.1)	*Bgl* II & *Hin*d III	909 (GI.1 P domain)
pMD19-P(GII.4)	*Bgl* II & *Eco*R I	948 (GII.4 P domain)
prTc-His C-*inaQ*	*Nco* I & *Bgl* II	525 (InaQN domain)

### Expression of recombinant plasmids

Recombinant *E. coli* BL21 strains were transformed with different recombinant plasmids, including pET28a-inaQn-ORF2(GI.1), pET28a-inaQn-ORF2(GII.4), pET28a-inaQn-P(GI.1), and pET28a-inaQn-P(GII.4), then cultured in Luria-Bertani (LB) (0.5% yeast extract, 1% trypton, and 1% NaCl) liquid medium containing 100 μg/mL kanamycin with shaking (150 rpm) at 37°C overnight. The transformed BL21 cells (50 μL) were transferred to 5 mL fresh LB medium (100 μg/mL kanamycin) with shaking (150 rpm) at 37°C. When the culture reached to optical density in 600 nm (OD_600_) of around 0.6, 0.4 mmol/L (final concentration) isopropyl β-D-1-thiogalactopyranoside (IPTG) was added and the cells were incubated at 16°C for 24 h with shaking (120 rpm). The cells were kept at 4°C for further use.

### Preparation of antibodies for VP1 of HuNoVs GI.1 and GII.4

#### Expression of recombinant capsid proteins in *E. coli*

*E. coli* BL21 with recombinant plasmids pET28a-ORF2 (GI.1) and pET28a-ORF2 (GII.4) were grown in LB medium with 100 μg/mL kanamycin with shaking (150 rpm) at 37°C overnight. The next day, the cells (2 mL) were subcultured in fresh LB (200 mL) with 100 μg/mL kanamycin with shaking (150 rpm) at 37°C. The recombinant plasmids were expressed in *E. coli* BL21 at 37°C for 4 h after induction by 0.4 mmol/L IPTG. All induced cells were collected for further use.

#### Purification of recombinant protein and preparation of specific antibodies

Recombinant capsid protein was purified according to previous report with minor modifications (Wang et al., 2008). Briefly, the cells expressing viral capsid proteins at the condition described in Section Expression of Recombinant Capsid Proteins in *E. coli* were washed 3 times using PBS (pH 7.2, NaCl 137 mmol/L, KCl 2.7 mmol/L, Na_2_HPO_4_ 10 mmol/L, KH_2_PO_4_ 2 mmol/L). The cells were resuspended in 20 mL Buffer A [pH 8.0, 50 mmol/L Tris-HCl, 1 mmol/L EDTA, 50 mmol/L NaCl, 5% glycerin (v/v), 0.5% Triton X-100 (v/v)] with 100 μg/mL lysozyme (Sigma, St. Louis, USA). After incubation at 37°C for 30 min, cells were subjected to ultrasonic treatment (BILON92-II, Shanghai, China) under the condition of 6 s with 4 s interval for 90 cycles on the ice. Then cell lysate was centrifuged at 10,000 × g for 15 min at 4°C. The precipitate (crude inclusion body) was washed by 10 mL Buffer A. Twenty milliliters lysis buffer (pH 9.0, 50 mmol/L Tris-HCl, 1 mmol/L EDTA, 50 mmol/L NaCl, 5% glycerin (v/v), 0.5% Triton X-100 (v/v), and 8 mol/L urea) was added to resuspend the precipitate and then resolved slowly on the ice for 2 h. After centrifugation at 10,000 × g at 4°C for 30 min, the supernatant was dialyzed in TE buffer (pH 8.0, 10 mmol/L Tris-HCl, 1 mmol/L EDTA) at 4°C for 12 h. The TE buffer was then replaced and continued second dialysis. After dialysis, the supernatant was centrifuged at 10,000 × g at 4°C for 30 min. The recombinant protein in supernatant was analyzed by SDS-PAGE according to our previous report (Wang et al., 2008).

#### Preparation of primary antibody against VP1

Ten female Balb/c mice (Hubei Provincial Center for Disease Control and Prevention, China), 5–6 weeks old, were immunized subcutaneously with 0.1 mg recombinant VP1 of HuNoV GI.1 or GII.4 every 2 weeks respectively. The sera were collected and used as primary antibodies against the recombinant viral capsid proteins (VP1 and P particles) after 3 months of immunization. The sera were stored at −80°C for further use.

#### Ethics statement

All animal studies were carried out in strict accordance with the recommendations in the Guide for the Institutional Animal Care and Use Committee. The protocol was approved by Laboratory Animal Feeding Standard Operation Procedure (SOP-Ani-019-2012), the Institutional Animal Care and Use Committee, and the Laboratory Animal Center of Shanghai Jiao Tong University (Permit Number: A2015020).

### Whole cell enzyme immunoassay (EIA)

A modified sandwich EIA was developed to evaluate the recombinant VP1 or P domains display efficiency on the surface of host cells with or without InaQN. Briefly, the transformed cells with recombinant plasmids pET28a-ORF2 (GI.1), pET28a-ORF2 (GII.4), pET28a-P (GI.1), pET28a-P (GII.4) and pET28a-inaQn-ORF2(GI.1), pET28a-inaQn-P(GI.1), pET28a-inaQn-ORF2(GII.4), pET28a-inaQn-P(GII.4), and plasmid pET28a as control were collected after induction of viral protein expression as described in previous Section Expression of Recombinant Plasmids by centrifugation at 5000 × g for 5 min. The pellet was washed with PBS (pH 7.2) twice and adjusted OD_600_ to around 1.0 in 500 μL PBS in a 1.5 mL Eppendorf tube. After centrifugation at 5000 × g for 5 min, the pellet was resuspended with 500 μL primary antibodies against VP1 obtained from Section Preparation of Primary Antibody Against VP1 (1:10,000 diluted in Tris-Buffered Saline and Tween-20 with 1% bovine serum albumin) and incubated for 30 min at 37°C. After washing with Tris-Buffered Saline and Tween-20 (TBST) for three times, the bacterial pellet was resuspended with 500 μL Peroxidase-Conjugated Goat Anti-Mouse IgG (H+L) (Yeasen, Shanghai, China) at a dilution of 1:5000 and incubated for 30 min at 37°C. After three washes with TBST, the bacterial pellet was resuspended in 500 μL PBS. Finally, a 50 μL of suspension was taken and mixed with 50 μL 3,3′,5,5′-tetramethylbenzidine [Friendbio Science and Technology (Wuhan) Co., Ltd., Hubei, China]. After keeping in the dark for 10 min, 50 μL of mixture was transferred to 96 well module (Lantian Biological Equipment Factory, Jiangsu, China), and 50 μL 2 mol/L H_2_SO_4_ was added to stop the reaction. OD_450_ value was measured using a Sunrise Microplate Reader (Tecan Sunrise, Switzerland). In addition, bacteria transformed with recombinant plasmids without *inaQn* gene including pET28a-ORF2 (GI.1), pET28a-ORF2 (GII.4), pET28a-P (GI.1), and pET28a-P (GII.4) were also tested.

### Measuring the HBGAs binding abilities

Type III PGM purchased from Sigma (St, Louis, MI, USA) was used as viral receptor. Each well of the Nunc Immuno Module (VWR, CA, USA) was coated with 100 μL of PGM (1 mg/mL in 0.05 mol/L carbonate-bicarbonate buffer, pH 9.6) at 4°C overnight. After being washed with PBS (pH 7.2) for 3 times, the wells were blocked with 120 μL of 1% bovine serum albumin (BSA) in PBS at 37°C for 1 h. The wells were washed with PBS (pH 7.2) for 3 times and used immediately.

The transformed bacteria were cultured as described in Section Expression of Recombinant Plasmids. The cells with recombinant plasmids pET28a-inaQn-ORF2(GI.1), pET28a-inaQn-P(GI.1), pET28a-inaQn-ORF2(GII.4), pET28a-inaQn-P(GII.4) were collected by centrifugation at 3000 × g for 5 min and diluted to 10^2^ or 10^3^ CFU/mL in PBS. The diluted cells (100 μL per well) were added into 48 wells, half of which were coated with PGM, and the remainder was not coated with PGM. After incubation at 37°C for 30 min, 10 μL suspension from each well was taken onto the LB agar plate containing 100 μg/mL kanamycin for overnight at 37°C. After incubation, the colonies were counted. In addition, recombinant plasmids without *inaQn* gene were constructed as a control including pET28a-ORF2 (GI.1), pET28a-ORF2 (GII.4), pET28a-P (GI.1), and pET28a-P (GII.4). The binding ratio was calculated by comparing the difference of colony forming units (CFU) before and after binding with or without PGM.

$$\begin{array}{l} \text{Binding ratio}=n/N\times 100\%\\ \;n=(\text{CFU in well without PGM})\\ \;-(\text{CFU in well with PGM})\\ \;N=\text{CFU in well without PGM} \end{array}$$

### Statistical analysis

IBM SPSS Statistics Software (version 19) was used for analyzing statistics. Each experiment was repeated at least three times (*N* = 3) as independent replicates in triplicates within each experiment (*n* = 3). One-way ANOVA was utilized for data analysis. Differences in means were considered significant when the *p* < 0.05.

## Results

### The fusion viral proteins expressed on the surface of transformed cells

**Figure 1:**
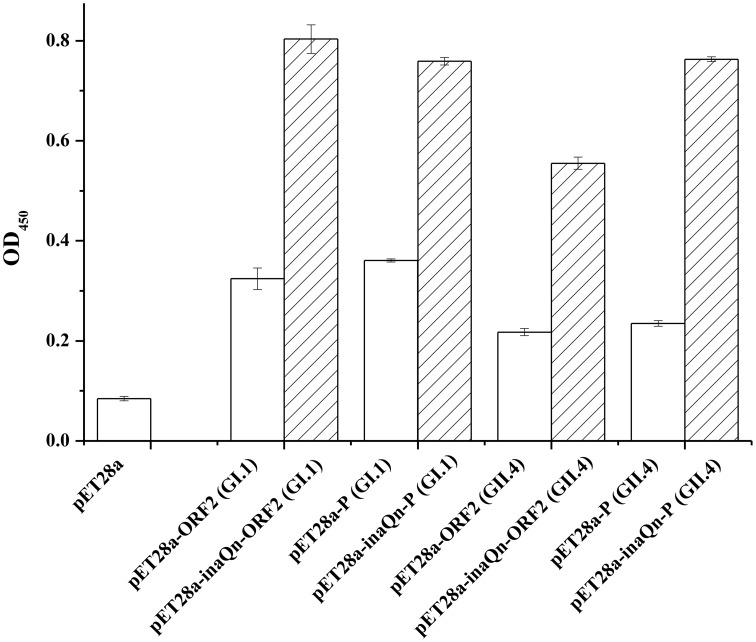
**Absorbance of 450 nm (OD_450_) in whole cell EIA assay**. The whole cell EIA results of recombinant HuNoVs VP1/P domains (□) and recombinant InaQN-HuNoVs VP1/P domains (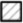). Bars represent standard error.

A modified sandwich EIA was developed to evaluate the recombinant VP1 or P domains display efficiency on the surface of host cells with or without InaQN (Figure 1). The results indicated the fusion protein could display on the surface of bacterial cells and maintain the antigenicity of HuNoVs (Figure 1). The absorbance was significantly higher in bacteria transformed with plasmids of *inaQn* gene fused with ORF2 or P domain coding gene than that of plasmids of ORF2 or P domain coding gene without *inaQn* gene (*p* < 0.01). The P/N (Positive result/Negative result) ratio of pET28a-inaQn-ORF2 (GI.1), pET28a-inaQn-P (GI.1), pET28a-inaQn-ORF2 (GII.4), and pET28a-inaQn-P (GII.4) were 9.45, 8.93, 6.53, and 8.98 respectively. Without the *inaQn*, the P/N ratio of pET28a-ORF2 (GI.1), pET28a-P (GI.1), pET28a-ORF2 (GII.4), and pET28a-P (GII.4) were 3.81, 4.25, 2.55, and 2.77 respectively. The presence of *inaQn* gene significantly increased display of HuNoVs capsid proteins on the surface of the transformed bacterial cells. There was no significant difference in signals of displayed VP1 and P domains of GI.1 transformed bacteria (*p* > 0.05). However, the signals of displayed P domains were significantly higher than VP1 in GII.4 transformed bacteria (*p* < 0.01). No significant difference was found among pET28a-inaQn-ORF2 (GI.1) (OD_450_ = 0.803 ± 0.029), pET28a-inaQn-P (GI.1) (OD_450_ = 0.759 ± 0.008), and pET28a-inaQn-P (GII.4) (OD_450_ = 0.763 ± 0.005) (*p* > 0.05). However, absorbance signal of pET28a-inaQn-ORF2 (GII.4) (OD_450_ = 0.555 ± 0.012) was significantly lower than the residual groups (*p* < 0.01).

### Binding of HBGAs with surface displayed VP1 and P domains

**Figure 2:**
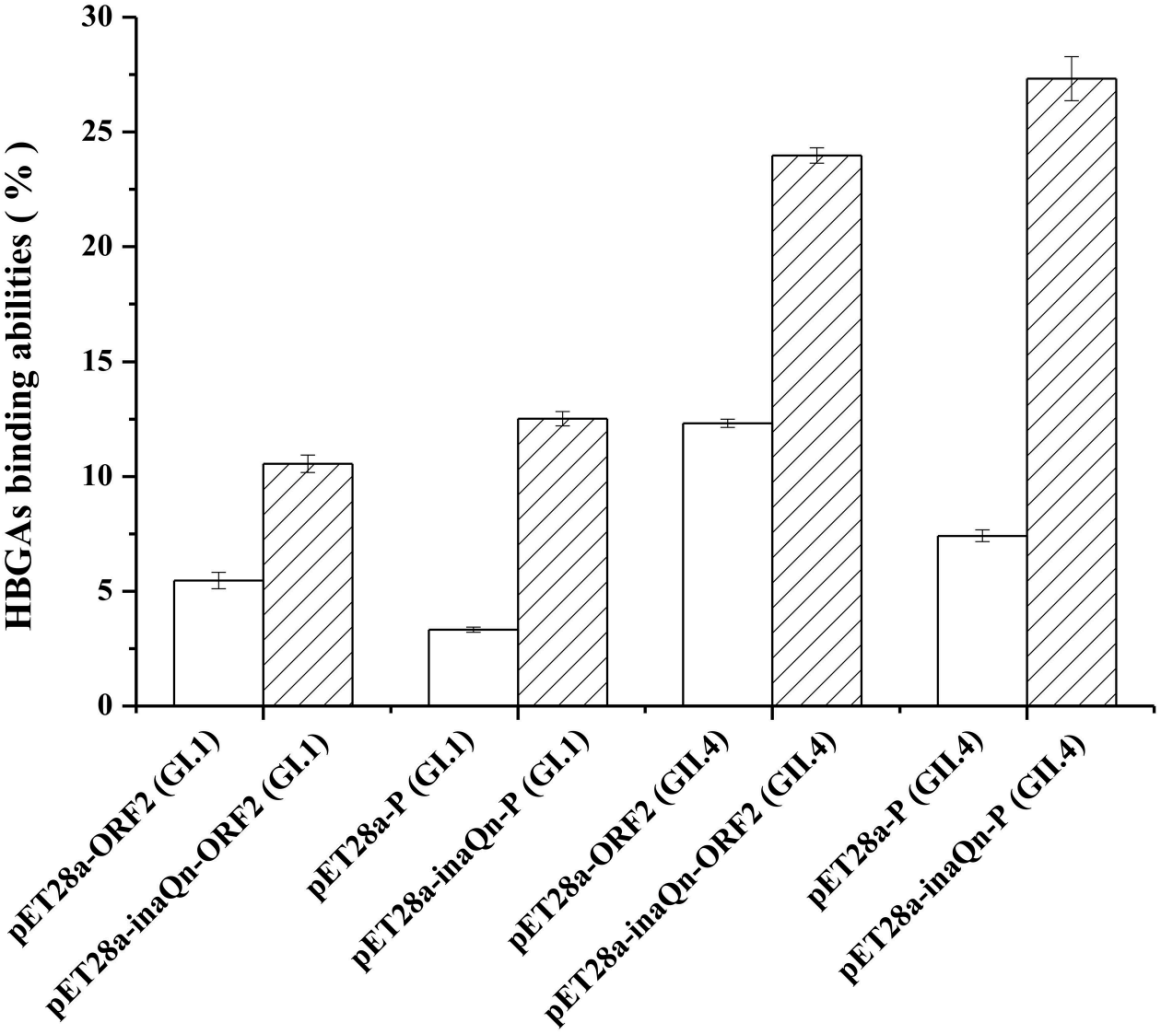
**HBGAs binding assay**. HBGAs binding abilities of *E. coli* with recombinant HuNoVs VP1/P domains (□) expression and recombinant InaQN-HuNoVs VP1/P domains expression (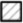). Bars represent standard error.

To identify the HBGAs receptor-binding capacity, *E. coli* BL21 cells were transformed with plasmids pET-28a, pET28a-ORF2 (GI.1), pET28a-inaQn-ORF2 (GI.1), pET28a-P (GI.1), pET28a-inaQn-P (GI.1), pET28a-ORF2 (GII.4), pET28a-inaQn-ORF2 (GII.4), pET28a-P (GII.4), and pET28a-inaQn-P (GII.4). The HBGAs binding capacity was evaluated by colony counting assay. When bacteria transformed with HuNoVs capsid protein encoding genes were added to PGM coated wells, the binding capacity between the HBGAs in PGM and displayed VP1 or P domains was determined by comparing the binding ratio of CFU in wells with or without PGM (Figure 2). The HBGAs binding capacity was 10.5 ± 0.4%, 12.5 ± 0.3%, 24.0 ± 0.3%, and 27.3 ± 0.9% in bacteria transformed with pET28a-inaQn-ORF2 (GI.1), pET28a-inaQn-P (GI.1), pET28a-inaQn-ORF2 (GII.4), and pET28a-inaQn-P (GII.4), respectively. The HBGAs binding capacity was 5.5 ± 0.4%, 3.3 ± 0.1%, 12.3 ± 0.2%, and 7.4 ± 0.3% in bacteria transformed with pET28a-ORF2 (GI.1), pET28a-P (GI.1), pET28a-ORF2 (GII.4), and pET28a-P (GII.4) without *inaQn*, respectively. Therefore, the HBGAs binding capacity of InaQN fused VP1s and P particles was significantly higher than groups without InaQN (*p* < 0.01). The HBGAs binding capacities of displayed P domains from both genotypes are slightly higher than displayed VP1. However, the HBGAs binding capacities of both displayed VP1 and P domains of GI.1 were significantly lower than that of GII.4.

## Discussion

In this study, cell surface display systems for HuNoVs capsid protein VP1 and P domains were constructed using InaQN as a carrier protein. To our knowledge, it is the first report on HuNoVs displayed the surface of transformed bacteria. Our results indicated that displayed VP1 or P domains of HuNoVs were successfully anchored on the surface of bacterial cells. The surface displayed VP1 or P domains maintained the antigenicity (Figure 1). The cell surface display using INP has been used in vaccine development. For example, a recombinant oral vaccine for hepatitis C has been tested (Lee et al., 2000). Therefore, this system could be a good candidate for vaccine development. Currently, we are in the process of using these cell surface displayed VP1 and P domains for vaccine candidate for HuNoVs.

The HBGAs have been recognized as receptors or co-receptors for HuNoVs (Huang et al., 2003). In this study, we demonstrated that the displayed viral proteins could interact with the HBGAs viral receptors (Figure 2). The HBGAs binding capacity of the displayed VP1 and P domains from GII.4 were significantly higher than displayed VP1 and P domains from GI.1, indicating GII.4 P domains or VP1 may fold more perfectly and be easily recognized by viral receptors than GI.1 P domains or VP1. Recently, sialic acids have been identified as additional cellular receptors/co-receptor for Tulane virus (Tan et al., 2015) and for HuNoVs (Rydell et al., 2009). It is possible that other receptors or ligands for HuNoVs have not been identified. VLP and P particles are limited for valuing in collection or isolation of HuNoVs receptors or ligands. VLP and P particles could not be used to get viral particle-ligand complex unless ultracentrifugation is applied. It is not practical to collect or isolate a viral particle-ligand complex by ultracentrifugation due to the small sample size. In this study, the results demonstrated that the viral capsid protein-receptor complex could be easily collected by low speed centrifugation. The cell surface display system we described in this paper could be an alternative method to replace the ultracentrifugation to collect the viral capsid binding ligands.

There was some background binding in bacteria expressing the viral capsid proteins (VP1 and P domains) without the *inaQn* gene. The background noise could be a result of non-specific binding of the viral capsid proteins on bacteria or nonspecific binding of the antibodies to bacteria as the antibodies were made from viral capsid proteins, which expressed in *E. coli* BL21. The background should be reduced significantly when the targets are viral receptors or ligands. Currently, we are in the process of isolating candidate ligands for HuNoVs using this approach.

This new strategy of using cell surface displayed HuNoVs capsid proteins will provide a new approach to isolate HuNoVs ligands, a new approach to characterize interaction between HuNoVs and receptors or ligands, a faster way to construct VP1 or P domains from a new strain or mutated strains. Since a couple of viral antigens displayed on the bacterial surface using INP have been proved to be an attractive platform for production of vaccine (Kim and Yoo, 1999; Kwak et al., 1999; Lee et al., 2000) and *E. coli*-expressed norovirus P particle could be a platform for antibody production (Tan et al., 2011), we believe that cell surface displayed HuNoVs capsid proteins can also be a good candidate for vaccine development.

## Author contributions

DW and MN designed the experiments. MN, QY, and DW carried out experiments. MN, DW, and XS analyzed sequencing data and experimental results. ZG provided HuNoV GII.4 clinical sample and analyzed the viral RNA sequence. PT provided plasmid pCR-TORO/GI.1-ORF2+3. MN, DW, and PT wrote and modified the manuscript. XS provided laboratory equipment and place.

### Conflict of interest statement

The authors declare that the research was conducted in the absence of any commercial or financial relationships that could be construed as a potential conflict of interest.
